# EphA2 in Cancer: Molecular Complexity and Therapeutic Opportunities

**DOI:** 10.3390/ijms252212191

**Published:** 2024-11-13

**Authors:** Lisa Toracchio, Marianna Carrabotta, Caterina Mancarella, Andrea Morrione, Katia Scotlandi

**Affiliations:** 1 Laboratory of Experimental Oncology, IRCCS Istituto Ortopedico Rizzoli, 40136 Bologna, Italy; lisa.toracchio@ior.it (L.T.); marianna.carrabotta@ior.it (M.C.); caterina.mancarella@ior.it (C.M.); 2 Sbarro Institute for Cancer Research and Molecular Medicine, Center for Biotechnology, Department of Biology, College of Science and Technology, Temple University, Philadelphia, PA 19122, USA; andrea.morrione@temple.edu

**Keywords:** EphA2, cancer hallmarks, targeted therapy, protein degradation, small molecule inhibitors, immunotherapy, nucleic-acid-based therapy

## Abstract

Erythropoietin-producing hepatocellular A2 (EphA2) is a member of the Eph tyrosine kinase receptor family that has been linked to various biological processes. In tumors, EphA2 overexpression is associated with noncanonical pathway activation, tumor progression, and a poor prognosis, which has emphasized its importance as a marker of malignancy. Studies on numerous cancer models have highlighted EphA2’s dual and often contradictory action, which can be attributed to EphA2′s interactions involving multiple pathways and different ligands, as well as the heterogeneity of the tumor microenvironment. In this review, we summarize the main mechanisms underlying EphA2 dysregulation in cancer, highlighting its molecular complexity. Then, we analyze therapies that have been developed over time to counteract its action. We discuss the limitations of the described approaches, emphasizing the fact that the goal of new options is high specificity without losing therapeutic efficacy. For this reason, immunotherapy or the emerging field of targeted protein degradation with proteolysis-targeting chimeras (PROTACs) may represent a promising solution that can be developed based on a deeper understanding of the molecular mechanisms sustaining EphA2 oncogenic activity.

## 1. Introduction

Receptor tyrosine kinases (RTKs) are a subclass of tyrosine kinases expressed at the plasma membrane regulating the transduction of biological signals between extracellular and intracellular compartments. In humans, there are 58 RTKs, categorized into 20 distinct families based on the composition of their extracellular domains [1]. Pathogenic RTKs mutations, deletions, translocations, and amplification/overexpressions are associated with the onset and progression of different types of human cancers. Accordingly, the inhibition of oncogenic RTKs using targeted therapy approaches such as multitarget or specific tyrosine kinase inhibitors (small molecules), antireceptor antibodies, or antiligand antibodies has revolutionized personalized treatment for cancer [1,2,3], particularly for some subgroups of cancer patients. Notable examples of successful anti-RTKs targeted therapy in routine clinical practice include anti-HER2 agents in metastatic breast cancer [4], anti-BCR–ABL agents in chronic myelogenous leukemia [5], and antiepidermal growth factor receptor (EGFR) therapy in colorectal cancer [6]. Other relevant responses have been obtained by using PDGFR/KIT inhibitors in gastrointestinal stromal tumors (GISTs) or by using anti-IGF-1R inhibitors in a percentage of Ewing sarcoma (EWS) patients [7,8]. There are factors limiting the clinical efficacy of targeted therapy, such as i. the molecular complexity surrounding each RTK, including intracellular and extracellular stimuli, which might contribute to mechanisms of acquired/intrinsic drug resistance; ii. lack of biomarkers of response/resistance; iii. kinase-independent, scaffolding nodes for RTKs oncogenic signaling. The ephrin (Eph) receptor family represents the largest family of RTKs [3] and drives crucial physiological processes, including cell sorting in embryo patterning, cell migration, growth cone retraction in axon guidance, formation of synaptic connections between neurons, blood vessel remodeling, and platelet aggregation [9,10,11,12,13,14]. In addition, Eph receptors play a critical role in several pathologies including cardiovascular diseases [15], viral infections [16], central nervous system diseases [17], and cancer [1,18]. Among Eph receptors, the role of EphA2 in different types of solid tumors, including carcinomas and sarcomas, is well established but it is often controversial, as EphA2 action can be pro- or antitumorigenic depending on ligand availability and/or cell/cancer model context. However, EphA2 is now considered an attractive target for therapy [19].

In this review, we will discuss the molecular mechanisms underlying the oncogenic functions of the Eph receptors family, focusing on the complexity of EphA2 in cancer and on how its unique features should be taken in account for an effective development and use of anti-EphA2 targeted therapies in cancer. We will also focus on how such complexity can impact responses to anti-EphA2 agents.

## 2. Eph System and Signal Transduction

In humans, the family of Eph receptors includes nine type A receptors (EphA1, EphA2, EphA3, EphA4, EphA5, EphA6, EphA7, EphA8, EphA10) and five type B receptors (EphB1, EphB2, EphB3, EphB4, and EphB6), based on their cognate ligand binding affinity. From a structural standpoint, Eph receptors are composed of 1. a multidomain extracellular region, with a ligand-binding (LBD), a Sushi, an EGF-like growth factor domain, and two fibronectin type III repeats; 2. a single transmembrane domain; 3. a juxta-membrane region; 4. a cytoplasmic domain containing the tyrosine kinase (TK) domain, the Sterile Alpha Motif (SAM) domains, a postsynaptic density protein (PSD)-95, Discs large, and a Zona Occludens tight junction protein (PDZ) binding motif [18]. The Eph family of ligands comprises ephrin A (five members) and ephrin B (three members), and the receptor/ligand interaction is mostly restricted within either the A or B family. A and B ephrins are characterized by a conserved Eph receptor-binding domain, which is connected to the plasma membrane by a linker region. The major difference between these two classes of ligands is in the intracellular portion, as type A ephrins are anchored to the plasma membrane by a glycosylphosphatidylinositol (GPI) anchor, while type B ephrins contain a transmembrane segment and a short cytoplasmic region.

The Eph system exhibits a series of peculiarities in its signal transduction pathway, rendering it exceptionally complex when compared to other RTKs [20,21]. The first peculiarity of the Eph system is the occurrence of bidirectional signals evoked by the ephrin/Eph interaction. Forward signaling is the typical signaling mode of RTKs and affects receptor-expressing cells, while reverse signaling affects ligand-expressing cells [18,22]. In the forward signaling mode, ephrin binding evokes the activation of Eph kinase activity, which results in the phosphorylation of tyrosine residues in the juxtamembrane domain, followed by oligomerization and clustering. Receptor activation leads to subsequent activation of intracellular effectors like Src family kinases, Ras Family GTPases, and p85 subunit of phosphatidylinositol 3′ kinase, while the AKT pathway can be suppressed [23]. Forward signaling is turned off by receptor endocytosis, trafficking into endosomes, and subsequent lysosomal/proteasomal degradation.

Reverse signaling is sustained by ephrins A and B through different intracellular mechanisms, according to the different structure of ligands. Receptor binding enables ephrin-B phosphorylation by kinases of the Src family, thereby creating binding sites for adaptor proteins such as Grb4 [23]. On the contrary, the mechanisms underlying ephrin A-mediated reverse signaling are still poorly understood. Data in the literature indicate that upon receptor binding, ephrin-A forms functional complexes with other RTKs such as TrkB [24] or Ret [25], thereby promoting downstream biological responses.

An additional level of complexity is the fact that the bidirectional (forward and reverse) signal can be activated via both trans and cis mechanisms. Trans Eph receptor–ephrin interaction occurs at intercellular junctions, when the receptor and ligands are expressed by adjacent cells, while cis interactions occur between Eph receptors and ephrins expressed by the same cell.

In addition to bidirectional signaling, another peculiarity of the Eph system is the processes of internalization and proteolytic cleavage. Similarly to other RTKs, following ligand-dependent activation, Eph receptors are endocytosed by Eph receptor-expressing cells, and degraded in either lysosomes or the proteosome. However, according to the “trans-endocytosis” process, Eph receptor–ephrin complexes can be also internalized by ligand-expressing cells. These processes are mostly dependent on the Rac pathway, particularly for EphB-ephrin B, proteolytic cleavage mediated by metalloproteases such as ADAM10 in the case of EphA-ephrin A [26], and ubiquitination mediated by the ubiquitin ligase c-Cbl [27,28]. In addition, the endocytic process can be modulated by mechanical and spatial stimuli, which depend on the interaction with other cells present in the microenvironment. In fact, the formation of high-density Eph-ligand clusters between neighboring cells inhibits the endocytic process and consequently alters downstream signaling. This then leads to the recruitment of metalloproteases, such as ADAM10, responsible for the dissociation of the ligand from the receptor. This process is essential for generating Eph repulsive responses which enable cellular deadhesion or retraction, fundamental for physiological cellular organization [29,30].

## 3. EphA2 in Cancer: Molecular Complexity and Impact on Cancer Hallmarks

The *EphA2* gene is located on chromosome 1p36 and encodes a 130 kDa protein of 976 amino acid, with a major role in physiological developmental processes in mammals including kidney development, bone homeostasis, mammary epithelial morphogenesis, and lens and inner ear formation [31]. However, aberrant activation of EphA2 has been documented in cancer initiation and progression in several human malignancies, including breast [32,33], prostate [34], bladder [35], colorectal [36], nasopharyngeal [37], lung [38,39], glioblastoma [40], ovarian [41], gastric [42], and melanoma [43] tumors as well as bone sarcomas, including osteosarcoma, EWS, and chondrosarcoma [44,45]. The notion that EphA2 plays a pivotal role in driving the progression of multiple cancers is also supported by a recent in silico pan-cancer study that used TCGA, GTEx, and CPTAC databases and analyzed EphA2 expression, tumor mutation, and DNA methylation status in normal versus tumor tissues as well as the association between EphA2 and clinical stage and prognosis in different tumors [46]. mRNA and protein expression levels of EphA2 were significantly upregulated in 15 out of 33 human tumors compared to normal controls. EphA2 expression levels significantly correlated with clinical stage of colon adenocarcinoma and testicular germ cell tumors, where EphA2 levels increased in advanced clinical stages, while in kidney renal cell carcinoma and ovarian carcinoma, EphA2 expression decreased. Considering EphA2 prognostic value, EphA2 expression correlated with risk factor and poor overall survival (OS) in 10 out of 33 neoplasia. Conversely, OS positively correlated with EphA2 expression in six cancers [46].

**Figure 1:**
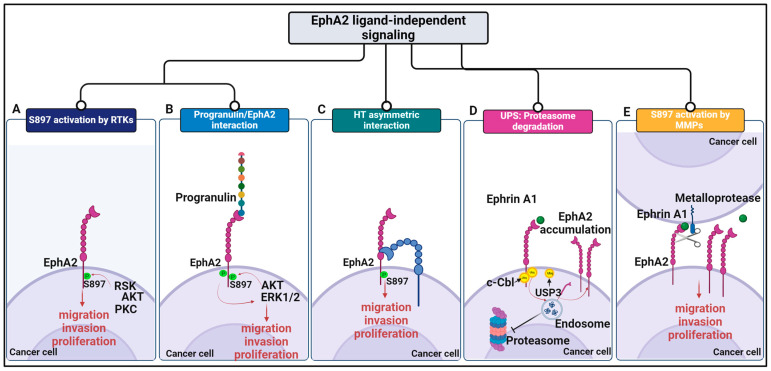
Schematic representation of EphA2-driven mechanisms in cancer. (**A**) In tumor cells, EphA2 predominantly acts through ligand-independent signaling and phosphorylation of the intracellular S897 residue by kinases that support proliferative and promigratory pathways. (**B**) An exception is the mechanism triggered by the interaction of EphA2 with progranulin, where this alternative ligand evokes EphA2 phosphorylation on S897 mediated prevalently by Erk1/2 and, to a lesser extent, AKT activation, driving a feedback mechanism on the receptor. (**C**) Ligand-independent homotypic head–tail (HT) interactions between the amino-terminal portion of EphA2 and the membrane-proximal domain of a neighboring receptor led to S897 activation and tumor progression. Panels (**D**,**E**) show two different ways to activate ligand-independent signaling. (**D**) The drawing illustrates the endocytosis of the ligand–receptor complex, followed by degradation. Alterations in the UPS/c-Cbl system are responsible for an imbalance in degradation mechanisms of EphA2, resulting in the accumulation of the receptor at the plasma membrane and the formation of clusters, which facilitates ligand-independent receptor activation. (**E**) EphA2 cleavage and processing by metalloproteases is a mechanism by which cancer cells can escape ligand-dependent tumor-suppressive signaling. Created using BioRender.

Collectively, these data support a dual role of EphA2 in cancer as EphA2 action can be either pro- or antioncogenic. Notably, only 3% of 10,967 TCGA pan-cancer patients showed EphA2 mutations, with missense mutations, amplification, and deletion being the predominant *EphA2* gene alterations [46], supporting the notion that the activity of EphA2 in cancer might predominantly rely on functional alterations of ligand/receptor interactions. In the canonical (ligand-dependent) pathway, EphA2 binds its natural ligand ephrin-A1, leading to autophosphorylation of tyrosine residues and activation of its intrinsic tyrosine kinase activity [31]. For most cancers, the main mechanism underlying EphA2 activity relies on its ligand-independent signaling, the so-called noncanonical pathway. This section will highlight data supporting major molecular mechanisms driven by EphA2 in cancer as well as the impact on cancer hallmarks that should be considered for effective anti-EphA2 therapy. The mechanisms discussed below are represented in Figure 1.

### 3.1. Noncanonical Phosphorylation of EphA2 S897: RTKs and Progranulin

In cancer cells, EphA2 overexpression is often coupled with low ephrinA1 expression [47,48]. Noncanonical EphA2 signaling is oncogenic and characterized by serine 897 (S897) phosphorylation modulated by three major signaling pathways: cAMP/PKA, AKT/mTORC1, and MAPK/RSK [11,49,50], and is associated with enhanced migratory and invasive capabilities of tumor cells as well as stemness and chemoresistance [51,52] (Figure 1A). For example, in glioblastoma, invasiveness of tumor cells is supported by ligand-independent, AKT-mediated phosphorylation of EphA2 S897, an event inhibited by ephrinA1 stimulation both in vitro and in vivo [51]. Accordingly, *Efna1*, *Efna3*, and *Efna4* triple-knockout mice, lacking the inhibitory effects mediated by canonical signaling, displayed increased invasive capabilities compared to wild type mice [51]. In addition, the EphA2/AKT axis promoted stem cell properties, as evidenced by the sustained expression of SOX2 and neurosphere formation [51]. Moreover, in ovarian cancer, RSK-mediated phosphorylation of EphA2 was associated with intrinsic and acquired chemoresistance to cisplatin and carboplatin [41]. Interestingly, inhibition or knockdown of RSK prevented EphA2-S897 phosphorylation, leading to a signaling shift to the canonical tumor-suppressive tyrosine phosphorylation and consequent downregulation of EphA2 [41].

Alternatively, EphA2 can be activated in tumors by non-ephrin ligands. One example is progranulin. As recently shown, progranulin is highly expressed in bladder cancer cells and is the prevalent ligand for EphA2, thereby constituting an oncogenic pathway driving motility, invasion, and in vivo tumor formation of bladder cancer cells [35,48]. The interaction between Progranulin and EphA2 triggers transient phosphorylation of Y588 on EphA2, which then activates AKT/ERK kinases, thereby generating a feedback mechanism promoting EphA2 phosphorylation at S897, driving tumor progression in bladder cancer [35] (Figure 1B).

### 3.2. Head–Tail Asymmetric Interactions

Another level of complexity associated with EphA2 action in cancer is its ability to form ligand-independent homotypic head–tail (HT) interactions between the amino terminus and the membrane proximal domain of neighboring EphA2 receptors. Shi et al. used time-resolved, live-cell fluorescence spectroscopy analyses, known as pulsed interleaved excitation–fluorescence cross-correlation spectroscopy (PIE-FCCS), to demonstrate that the ectodomain of EphA2 can form symmetric head–head (HH) interactions when bound to ephrin A1 ligands [53]. However, in a ligand-free state, EphA2 presents with asymmetric homotypic HT interactions, which cause ligand-independent phosphorylation of S897 and oncogenic signaling in cancer cells. HT interactions generate signals associated with in vitro migration and in vivo tumor invasiveness, as shown by functional studies performed in different tumor models, including prostate cancer and skin carcinoma [53] (Figure 1C).

### 3.3. Proteasomal and Lysosomal EphA2 Degradation

Data from the literature demonstrate that the ubiquitin proteosome (UPS) machinery plays a significant role in modulating EphA2 protein stability, with relevant implications on EphA2 action in tumor cells. The UPS system represents a major intracellular regulator of protein stability, homeostasis, and trafficking. In UPS, ubiquitin-activating enzymes (E1), ubiquitin-conjugating enzymes (E2), ubiquitin-protein ligases (E3), and deubiquitinating enzymes cooperate to drive ubiquitinated proteins to the proteasome for degradation [54]. The E3-ligase c-Cbl acts as a negative regulator of EphA2 as it induces proteasomal degradation of ephrin-A1-activated EphA2 [55,56,57] (Figure 1D). Notably, Ola Sabet and colleagues identified tyrosine 813 on EphA2 as the docking site for c-Cbl [56] and demonstrated that ligand-induced activation of EphA2 promoted EphA2 clustering, phosphorylation of a c-Cbl docking site, and ubiquitination of the receptor, which sorted EphA2 into late endosomal/lysosomal compartments, thereby promoting receptor degradation [56]. From the functional standpoint, c-Cbl-mediated ubiquitination of EphA2 represents a crucial oncosuppression mechanism in cancer cells [57].

However, c-Cbl is not the only ligase responsible for mediating EphA2 ubiquitination. Mass spectrometry analysis demonstrated that the E3 ligase RNF5 directly interacted with EphA2 and induced EphA2 ubiquitination and degradation in HER2-negative breast cancer cells [32], thereby suppressing EphA2 tumor-suppressive functions in this neoplasia. Accordingly, RNF5 depletion using shRNA approaches or inhibition using the INH2 compound increased cell surface EphA2 levels, decreased ERK and AKT phosphorylation, and altered the balance of EphA2 phosphorylation at S897 and Y772, resulting in a tumor-suppressor function. Overall, RNF5 modulates EphA2 level and tumor-suppressor function to facilitate tumor formation of HER2-negative breast cancers [32].

Other E3 ligases involved in the degradation of EphA2 include UBE4A [58]. Mechanistically, UBE4E interacts with the Src-like adaptor protein (SLAP), which belongs to the subfamily of hematopoietic adaptors that inhibit intracellular signaling. The SLAP–UBE4E interaction induces EphA2 ubiquitination and proteasomal degradation, therefore hindering EphA2 oncogenic signaling in tumor cells [58]. On the contrary, deubiquitinating enzymes, such as the ubiquitin-specific protease 3 (USP3), deubiquitinate EphA2 at K882 and K945 sites, leading to EphA2 protein stabilization [59]. As shown in osteosarcoma cells, overexpression of USP3 decreased EphA2 ubiquitination and stabilized EphA2 protein, whereas USP3 depletion enhanced ubiquitin-mediated EphA2 degradation. In addition, USP3 promoted proliferation and metastasis of osteosarcoma cells by enhancing in vitro EphA2-mediated PI3K/AKT signaling pathway and in vivo tumor growth [59].

### 3.4. Proteolytic Cleavage by Metalloproteases

In cancer cells, EphA2 action is the result of a delicate balance between oncosuppressive and oncogenic signals, which depend on the availability of cognate ephrin ligands. Cancer cells very often express EphA2 ligands, including ephrinA1, which induces EphA2 Y588-phosphorylation in brain, breast, and cervical cancer [35,60]. EphA2 cleavage and processing by metalloproteases is a mechanism by which cancer cells can escape ligand-dependent tumor-suppressive signaling (Figure 1E). Accordingly, it has been reported that in metastatic breast carcinoma, simultaneous overexpression of EphA2 and membrane-anchored membrane type-1 matrix metalloproteinase (MT1-MMP) leads to the cleavage of the fibronectin III domain of EphA2 and consequent loss of its ligand binding site. This event is modulated by EphA2-dependent Src activation, which is necessary for MT1-MMP cleavage, and determines EphA2 internalization, cellular repulsion, and single-cell invasion in 3D models sustained by RAS/ERK1/2, PI3K/AKT, and RhoG/ELMO-2/DOCK-4/Rac-1 signaling pathways [61,62].

Significantly, in another work, Koshikawa et al. showed that an engineered cleavage-resistant form of EphA2 reconstituted canonical signaling, thereby inhibiting tumor growth and metastasis formation in human tumor xenograft models [63]. In addition, other data indicate that modulating the cleavage of ephrinA1 ligand can also have an impact on the oncogenic activation of the EphA2 axis. As recently demonstrated, the metalloprotease ADAM17 contributed to the release of ephrin-A1 from the cell surface of non-small-cell-lung (NSCL) cancer cells, thereby enhancing EphA2-S897 phosphorylation, and ADAM17 activity was associated with enhanced in vitro cancer cell migration and resistance to ionizing radiation [38].

### 3.5. EphA2 Oncogenic Cross-Talk with the Microenvironment

Extracellular stimuli from the tumor microenvironment plays an important role in modulating EphA2 oncogenic function [64,65].

For instance, the interaction of EphA2 with Caveolin-1 (CAV1), an integral membrane protein, leads to the formation of new vessels in the tumor microenvironment of EWS cells [66]. In EWS cells, EWS::FLI1 functions as an oncogene because its acts as an aberrant transcription factor [67]. As such, the oncogenic effect of EWS::FLI1 is closely related to genes it directly regulates, including *VEGF* and *CAV1* [68]. In EWS tumor, EphA2 interacts with CAV1 through a caveolin binding motif (WSYGIVMW). This interaction is crucial for receptor active conformation, which stimulates AKT activation and subsequent bFGF production, thereby promoting angiogenesis [68]. Accordingly, inhibition of CAV1 in mice reduced vascular density and tumor growth as well as in vitro inhibition of EphA2. Specifically, CAV1 depletion impaired EphA2 tyrosine phosphorylation, leading to receptor internalization and subsequent degradation associated with AKT inhibition, downregulation of bFGF, and inhibition of motility [68]. It is well established that the Eph/ephrin system regulates vascular formation and remodeling. Specifically, EphA2 expression is elevated in angiogenic vasculature compared to quiescent, postnatal vessels [69]. EphA2 regulates angiogenesis and vascular permeability in synergy with the ligand Ephrin A1, as well as with a range of endothelial growth factors including VEGF. This evidence positions EphA2 as an attractive target for antiangiogenic therapies [69]. The role of EphA2 in sustaining neoangiogenesis was also reported in ovarian carcinoma, where EphA2 was associated with high expression of vascular endothelial growth factor (VEGF-a) and vascular endothelial cadherin (VE-cadherin) upregulation at both mRNA and protein levels [70]. EphA2/VE-cadherin regulated the activity of matrix metalloproteinases, including MMP9, which promoted cleavage of laminin 5 γ2-chain into promigratory γ2′ and γ2x fragments [70]. The release of these fragments in the tumor microenvironment increased the formation of vessels through a process known as vascular mimicry (VM) [71]. The association between EphA2 and VEGF also plays an important role in modulating drug resistance. EphA2 drives resistance to the VEGF-a-targeting monoclonal antibody bevacizumab, as a study conducted in glioblastoma patients revealed that patients who developed resistance to bevacizumab had high expression of EphA2, as well as proangiogenic factors such as FGF2, and AGPT2 [71]. Recent studies have shown that EphA2 can additionally support tumorigenesis by promoting tumor immune evasion. For example, in melanoma, the invasion depth of tumor lesions correlated with increased expression of genes like *EphA2,* promoting evasion from the immune system and preventing recruitment of T lymphocytes [72]. Recently published data demonstrated that endogenous lactate dehydrogenase A (LDHA) was upregulated in renal carcinoma cells and promoted EphA2 overexpression in cancer-derived exosomes, which promoted M2 macrophage polarization through the activation of the PI3K/AKT/mTOR and tumor progression [73].

## 4. Complexity of EphA2 Targeting in Cancer

Based on the preclinical and translational evidence supporting a specific role of EphA2 in cancer, different approaches have been developed and tested in recent time to block its activity in tumor cells. However, targeting EphA2 in cancer requires a deep understanding of its dual function and the intrinsic complexities of EphA2 signaling pathways. In the following sections, we will cover available anti-EphA2 therapeutic approaches to highlight specific limitations as well as advantages of each discussed strategy.

### 4.1. Inhibiting EphA2 Using Small Molecules

Various small molecules targeting EphA2 have been developed, and they can be divided into two major classes of inhibitors. Protein–protein interaction (PPI) inhibitors target the EphA2 LBD and interfere with the interaction between EphA2 and ephrin ligands, thereby affecting EphA2 phosphorylation [40,74]. Kinase domain inhibitors instead include ATP-mimicking agents, which block EphA2 kinase activity and downstream signaling [75]. PPI inhibitors targeting EphA2 LBD have a wide range of chemical structures spanning from salicylic acids to various bile acid derivatives as well as other derivative compounds repurposed to block EphA2. In addition, this class of inhibitors includes both EphA2 agonists and antagonists, which simulate or prevent EphA2/ligand interactions, respectively. Overall, the large receptor/ligand interaction surface of EphA2 poses major challenges in designing effective PPI inhibitors. Among agonists, which are designed to recover tumor-suppressing ligand-dependent activity of EphA2, the small molecule doxazosin showed good preclinical results in in vitro and in vivo cancer models [76]. Doxazosin successfully inhibited AKT and ERK kinase activities in an EphA2-dependent manner, triggered EphA2 receptor internalization, suppressed migration of prostate, breast, and glioma cancer cells, reduced distal metastasis of human prostate cancer cells, and prolonged survival in mice [76]. In another study on NSCL, doxazosin exhibited inhibitory activity on VM by suppressing the transcription of VM-related genes such as *VEGF-A*, *MMP-2*, and *VE-cadherin*. Consequently, the downstream EphA2/AKT/mTOR/MMP/Laminin-5γ2 pathway was significantly downregulated in response to doxazosin treatment [77]. Further chemical optimization of doxazosin led to the identification of other compounds with unique dimeric structures and superior activity compared to doxazosin, including the capability of crossing the blood–brain barrier in preclinical models [78,79]. Despite promising preclinical results, all these agents still require further optimization before being tested in clinical trials.

Several compounds have been repurposed to block EphA2 action as EphA2 antagonists, including Farnesoid X Receptor (FXR), GW4064, cilofexor, nidufexor, tropifexor, turofexorate isopropyl, and vonafexor, which demonstrated good preclinical results in tumor models, including prostate cancer [80]. However, while some of these FXR agonists are currently in clinical trials, there are no trials yet assessing their efficacy specifically in EphA2-positive tumors. In addition to natural ligands, synthetic agents, including chimeric proteins like soluble EphA2 or ephrin-A1, and peptides, can be used to target the Eph-ephrin system. These agents recapitulate the antitumor effects observed when ephrinA1 binds to EphA2 [81].

Inhibitors that specifically target the EphA2 kinase domain are limited, as most of the drugs available inhibit EphA2 but lack specificity for this target. These compounds have different chemical structures including catechol and quinazoline derivatives, as well as nanomolar inhibitors such as ALW-II-41-27 and GLPG1790. For instance, ALW-II-41-27 is a widely used inhibitor of EphA2 kinase activity in preclinical models, including bone sarcomas, and shows the ability to inhibit cell growth of different neoplasia [44,82]. However, it shows cross-reactivity with other kinases such as b-raf, CSF1R, DDR1, DDR2, EphA5, EphA8, EphB1, EphB2, EphB3, Frk, Kit, Lck, p38α, p38β, PDGFRα, and PDGFRβ [83], evoking the risk of severe off-target effects. On the contrary, the tyrosine kinase inhibitor dasatinib, originally designed to target BCR/ABL and Src family kinases, displays high affinity for EphA2 as off-target effect, and preclinical studies demonstrated that it is particularly active in tumors with high expression of oncogenic S897-phosphorylated EphA2 [84]. Accordingly, a clinical study of dasatinib plus paclitaxel and carboplatin showed promising clinical activity with manageable toxicity in endometrial cancer [85]. Ledipasvir (LDV) and daclatasvir (DCV), two antiviral molecules, inhibit AKT phosphorylation by destabilizing the Src/EphA2 complex. Mezquita et al. demonstrated that LDV and DCV are particularly effective in inhibiting proliferation, invasion, and colony formation in triple-negative MDA-MB-231 breast cancer cells, SRC-transduced SW620 colon cancer cells, and SRC-transduced NIH3T3 fibroblasts, suggesting potential therapeutic applications in Src-associated tumors [86].

### 4.2. EphA2 Targeting Using Monoclonal Antibodies

**Figure 2:**
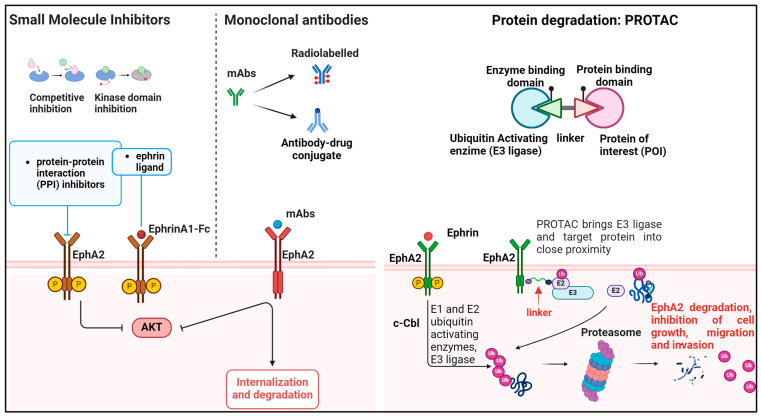
Schematic representation of EphA2 inhibition versus degradation approaches in cancer. Tyrosine kinase inhibitors, small molecules, or monoclonal antibodies inhibit EphA2 either by mimicking the action of ephrin A1 ligands and activating canonical signaling, or by inhibiting the kinase domain. mAbs can be also conjugated to drugs or toxins that target the internalization and degradation of the receptor. PROTACs are next-generation constructs that respond to the demand for greater specificity in receptor recognition. Their action is driven by linker molecules and the ubiquitin system, which trigger targeted protein degradation, resulting in the inhibition of cell growth, migration, and invasion. Created using BioRender.

Monoclonal antibodies (mAbs) targeting EphA2 have a dual mechanism of action, as they not only mimic the function of the cognate ligand ephrin A1 to induce EphA2-dependent antioncogenic responses, but also downregulate EphA2 protein levels by inducing internalization and degradation, thereby inhibiting EphA2 signaling (Figure 2). EA1.2, EA2, or B233 were among the first generated anti-EphA2 mAbs and showed efficacy in preclinical studies by inhibiting cancer cell growth both in vitro and in vivo [87,88]. Several mAbs were then developed such as SHM16 [89], DS-8895a [90], and MEDI-547 [91], and showed promising preclinical results in different cancer models. Anti-EphA2 antibodies are characterized by high specificity for EphA2 as compared to small-molecule inhibitors. Accordingly, EphA2 antibodies were tested in phase I clinical trials, but the overall results indicated poor efficacy and excessive drug toxicity. Two phase I trials were conducted in patients with advanced or metastatic EphA2-positive cancers to test safety, tolerability, pharmacokinetics, pharmacodynamics, and biodistribution of the anti-EphA2 mAb DS-8895a [92,93]. DS-8895a was safe and well tolerated, but it showed limited therapeutic efficacy, with no or limited objective tumor response. Lack of efficacy was attributed to low DS-8895a tumor uptake, as assessed using radio-labeled conjugate 89Zr-DS-8895a. This response might possibly reflect the heterogeneity of EphA2 expression and/or low expression of EphA2 in metastatic lesions [93]. In addition, lack of response could be due to the underestimation of the molecular complexity of the EphA2 system, thereby supporting the use of combinatorial treatment strategies rather than single-agent approaches. For instance, DS-8895a treatment was associated with decreased expression of the immune checkpoint inhibitor (ICI) PD-L1, suggesting the possible benefits of DS-8895a in association with ICIs [92].

MEDI-547 is an antibody–drug conjugate (ADC) composed of a human immunoglobulin (Ig) G1 monoclonal antibody directed against EphA2 (known as 1C1) and conjugated on cysteine residues to an auristatin derivative linker-toxin maleimidocaproyl-monomethyl auristatin phenylalanine, intended for delivering chemotherapy drugs directly to EphA2-expressing cancer cells. MEDI-547 shows strong in vitro and in vivo antitumor activity. Preclinical studies indicated that treatment with MEDI-547 induced EphA2 degradation and internalization, decreased proliferation, increased apoptosis, and displayed antiangiogenic effects in endometrial and prostate cancer cells and mouse and rat orthotopic models [91,94]. Based on sound preclinical results, a phase I, open-label study of MEDI-547 was conducted in patients with relapsed or refractory solid tumors [95]. However, the study was interrupted due to treatment-related toxicity, along with disease progression in patients, thereby not supporting further clinical studies. Notably, the most frequently reported treatment-related adverse events were bleeding and coagulation events as well as increased liver enzymes, decreased hemoglobin, decreased appetite, and epistaxis, while a minority of patients experienced serious adverse events, including conjunctival hemorrhage, pain, liver disorder, and hemorrhage [95].

More recently, the auristatin-based hSD5-vedotin antibody–drug conjugate targeting EphA2 was tested in a preclinical setting [96], and in pancreatic cancer, hSD5-vedotin triggered EphA2 endocytosis, strongly inhibited in vitro tumor growth and promoted apoptosis as well as suppressed tumor growth in pancreatic cancer xenograft animal models [96]. However, whether this compound is clinically safe is not yet defined.

### 4.3. Targeting EphA2 Using Protein Degradation

The possibility of modulating protein stability represents a valuable therapeutic modality to block oncogenic effectors in cancer [97]. Different therapeutic strategies are currently under development for inhibiting or exploiting the UPS system. FDA-approved agents for clinical use in cancer patients include inhibitors of E3 ligases, proteasome, or deubiquitinating enzymes [98]. More recently, there is a lot of interest in targeted protein-degradation-based strategies using “degraders”, including PROTACs and molecular glues, which induce forced proximity between protein-of-interest (POI) and ubiquitin ligases, driving proteasomal-mediated degradation of therapeutic targets [99] (Figure 2).

As previously mentioned, the E3 ubiquitin ligase c-Cbl acts as negative regulator of EphA2 action by mediating ephrinA1-dependent ubiquitination and proteasomal degradation of the receptor [55,56,57]. Accordingly, inhibition of the c-Cbl-EphA2 interaction, due to molecular competitors such as the Ca2^+^ or phospholipid-binding protein Annexin A1 (ANXA1), increases EphA2 protein stability and promotes nasopharyngeal carcinoma in vitro and in vivo growth and metastasis [57]. From a therapeutic standpoint, the use of an ANXA1-derived 11 amino acid–long peptide (named A11) which occupies the ANXA1-binding site on EphA2, preventing its binding to EphA2, increased c-Cbl interaction with EphA2, receptor ubiquitination, and degradation, thereby inhibiting in vitro cell growth, migration, and invasion, as well as in vivo tumor growth [57]. Similarly, the E3 ligase RNF5 directly interacted with EphA2 and induced EphA2 ubiquitination and degradation in HER2-negative breast cancer cells [32], thereby suppressing EphA2 tumor-suppressive functions in this neoplasia. For therapeutic purposes, RNF5 inhibitors are available, including the recently developed inhibitor and degrader compound FX12, which promotes RNF5 protein degradation through proteasomal-dependent endoplasmic reticulum (ER)-associated degradation pathway [100]. Thus, FX12 might be useful in counteracting tumor growth in those cancers where EphA2 has a tumor-suppressive action. Recent evidence indicates that EphA2 might be sensitive to proteasomal degradation by PROTACs [101,102]. In the field of targeted protein degradation, PROTACs are heterobifunctional molecules combining a target-binding warhead, a linker, and a E3 ligase-recruiting moiety. Despite the challenges in the chemical development of these agents, targeted protein-degradation-based modalities are promising for overcoming limitations of classical targeted therapies. Compared to therapies against RTKs, PROTACs allow us to target proteins lacking a kinase domain or block specific protein isoforms, in this way counteracting the onset of acquired drug resistance [103]. Different PROTACs are currently being tested in clinical trials [99]. The PROTAC2 compound has a small molecule foretinib, an ATP-competitive multiple RTKs inhibitor [104], as target-binding warhead, and a moiety recruiting the E3 ligase cerebron. It showed high binding affinity for EphA2 and c-MET. Accordingly, PROTAC2 induced in vitro protein degradation of EphA2 and c-MET [102]. Even though these results are still very preliminary and the functional impact of PROTAC2-mediated degradation of EphA2 in tumor cells is still unexplored, PROTAC may represent a concrete opportunity for targeting EphA2.

A schematic representation of the discussed approaches is shown in Figure 2.

### 4.4. EphA2 Bicycle-Based Therapy in Cancer

Bicyclic peptides are currently being explored as novel approaches for treating cancers resistant to traditional therapies such as chemotherapy or monoclonal antibodies.

**Figure 3:**
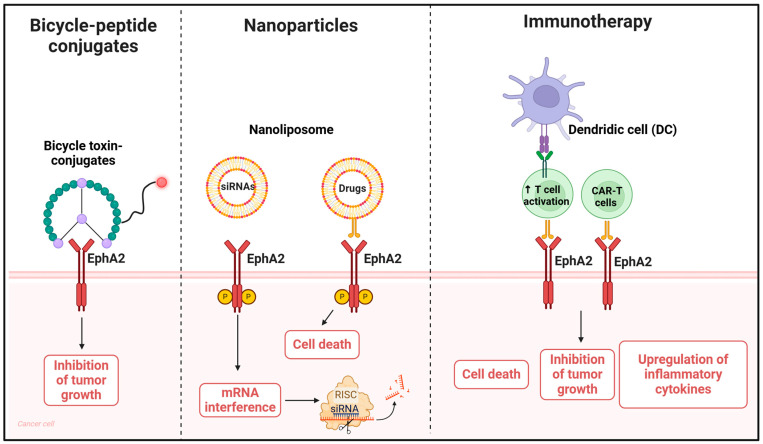
Schematic representation of bicycle peptides, nanoparticles- or immunotherapy-based therapeutic strategies targeting EphA2. Bicycle peptides can deliver toxic conjugates, while nanoparticles can be a useful strategy for delivering drugs or siRNAs directly to tumor cells with high EphA2 expression. Nanoliposomes carrying siRNAs against EphA2 downregulate receptor expression. Conversely, peptide-conjugated nanoliposomes targeting EphA2 exploit the presence of the receptor on tumor cells to allow the direct and specific internalization of drugs in cells with high EphA2 expression only. In immunotherapy, two approaches can be used: recruitment of killer cells by T cells engineered to recognize tumor cells expressing EphA2, or CAR-T cells, with the common goal of inducing tumor cell death. Created using BioRender.

These peptides are small, engineered proteins with a bicyclic structure, which provides enhanced stability, high specificity, and strong binding affinity to targets [105]. Bicycle toxin conjugates (BTCs), being very small compounds, offer the advantage of faster and improved tumor penetration compared to traditional ADCs (Figure 3). Any conjugates not bound to the tumor are swiftly eliminated, minimizing toxicity for healthy tissues [105]. Recently, Mudd G. et al. identified through screening analysis BT5528, an EphA2-targeting BTC, which binds, at low nanomolar range, the ligand binding motif of EphA2 through a cleavable linker. The authors compared MEDI-547, an ADC, with BT5528, evaluating efficacy, pharmacokinetics, and toxicity. The study revealed that although both compounds load the same MMAE payload, BTCs showed less toxicity in normal tissues [106]. Moreover, the efficacy of this compound showed excellent results in terms of dose tolerability in rats, and clinical evaluation is currently underway in advanced solid tumors overexpressing EphA2 (NCT04180371) [107].

### 4.5. EphA2-Based Nanoparticles in Cancer Therapy

Several preclinical and clinical studies have recently explored the potential of EphA2-targeted nanoparticles. The aim of this approach is to improve targeting efficacy, specificity, drug loading capacity, and stability and safety of these nanoparticles. EphA2-targeted nanoparticles are, in most cases, liposomes, i.e., spherical vesicles with a lipid bilayer that can be loaded with small interfering RNAs (siRNAs) or drugs for targeted delivery to cancer cells, or polymeric nanoparticles made from biodegradable polymers [108,109]. RNA interference (RNAi)-based therapy is an effective tool for gene silencing. SiRNA or short hairpin RNA (shRNA) can be designed to specifically target EphA2 mRNA, leading to its silencing and reduced protein levels. In glioma cells, EphA2 inhibition by siRNA approaches decreased tumor cell proliferation and induced apoptosis [110]. Despite the in vitro efficacy of this approach, in vivo delivery of siRNAs poses a great challenge and constitutes the major limitation for its clinical use [111,112,113]. Nanoparticles delivery systems are designed for overcoming this challenge. SiRNA-loaded liposomes are generally internalized via clathrin-mediated endocytosis, macropinocytosis, and cholesterol-dependent pathways [114]. For example, liposome-incorporated EphA2 siRNA (EPHARNA), which uses liposomes composed of the neutral lipid 1,2-dioleoyl-*sn*-glycero-3-phosphatidylcholine (DOPC), showed some efficacy in in vivo orthotopic models [115]. Moreover, this system was assessed in Rhesus monkeys and the results obtained by these experiments demonstrate that EPHARNA was well tolerated at all doses tested and elicited therapeutic efficacy without significant toxicity [116]. Collectively, these data suggest that EPHARNA may represent a novel method for therapeutic delivery of siRNAs, and a human phase I clinical trial is currently underway (NCT01591356) [116]. With a similar approach, cationic solid lipid nanoparticles (cSLNs) were designed to cargo anti-EphA2 siRNAs in prostate cancer. As already mentioned above, the use of nanoparticles was necessary to overcome poor stability and low uptake of siRNAs, thereby providing an effective delivery system [117].

In another work, the authors developed EphA2-targeted nanoliposomes loaded with doxorubicin (DOX) and siRNAs against JNK-interacting protein 1 (JIP1). Dual treatment with DOX-siRNA JIP1 was effective in restoring chemosensitivity to DOX, but these agents showed poor cellular uptake and high instability in plasma [118]. DOX-siRNA JIP1 loading on EphA2-targeted carriers were very effective in inducing toxicity of osteosarcoma cells, which exhibit high level of EphA2 expression [118]. More recently, microvesicles (MVs) coated with surface-carboxyl Fe_3_O_4_ superparamagnetic nanoparticles (SPIONs) conjugated with EphA2-targeted peptides (YSAYPDSVPMMS, YSA) were designed and used to treat osteosarcoma. The use of YSA-SPION-MV/MTX in murine models allows for the specific delivery of drugs to the tumor site, with great efficacy and low toxicity compared to MTX treatment alone [119]. In another study, a novel EphA2-targeting antibody-directed nanotherapeutic, encapsulating a labile prodrug of docetaxel (EphA2-ILs-DTXp), was developed to treat EphA2- expressing bladder cancers [120]. EphA2-ILs-DTXp was particularly effective in four EphA2-positive patient-derived xenograft (PDX) models of the disease, especially compared to monotherapy with docetaxel. Additionally, the study demonstrated that the combination with gentamicin had synergistic effects in inhibiting tumor growth in these PDX models [120].

Despite recent advances in chemotherapy, systemic drug delivery is not always successful due to poor uptake at the tumor site or toxicity associated with treatments. Thus, more specific delivery systems are needed to increase treatment efficacy and survival rate in patients. To address this need, several studies have been conducted for developing more specific targeting carriers and minimizing side effects. In lung cancer, pegylated EphA2 peptide-coated nanoparticles simultaneously delivered two therapeutic agents with high affinity for lung tumor cells expressing EphA2. In addition to target specificity, these nanoparticles showed high in vivo tumor tissue penetrance with enhanced antitumor activity and reduced toxicity [121]. In breast cancer, EphA2 overexpression was exploited to more precisely deliver the therapeutic agent DOX using mesoporous silica nanoparticles (MSNs) modified with the YSA peptide recognizing EphA2. These nanoparticles increased therapeutic efficacy with low toxicity in EphA2-overexpressing breast cancer cells [122].

### 4.6. EphA2 and Immunotherapy

EphA2 represents an important antigen that can be used for immunotherapy-based strategies, as EphA2 immunotherapy represents a promising frontier in cancer treatment, with ongoing research aimed at optimizing efficacy and safety.

Cancer vaccines are designed to stimulate patients’ immune systems and induce a response against EphA2-expressing tumor cells. Vaccines can be based on dendritic cells (DCs), a leukocyte population, which induce both cytotoxic T lymphocytes (CTLs) and helper T cells activation [123]. In this context, DC-based vaccines pulsed with EphA2 peptides were very effective in eliciting antitumor immune response in murine models of colorectal cancer [124]. Moreover, recent research has explored novel strategies with ICIs therapy to enhance DC-based vaccines’ immune response. Currently, two pilot clinical trials are recruiting patients with locally advanced solid metastatic tumor or with refractory/relapsed lymphomas to assess the efficacy and safety of EphA2-DC-based vaccines in combination with ICIs (NCT05631886; NCT05631899).

Another strategy is based on chimeric antigen receptors (CARs)-modified T (CAR-T) cell therapy. T cells are genetically modified to recognize specific antigens targeting tumor cells [125]. Notably, EphA2-CAR-T cells were developed and tested on glioblastoma, where these cells recognized and suppressed EphA2-positive glioblastoma cells with significant reduction in in vivo tumor growth [126]. Similar results were also obtained in NSCLC, xenograft mouse models of lung cancer [127], and immunotherapy of esophageal squamous cell carcinoma [128]. A clinical trial was initiated to evaluate the safety and efficacy of CAR-T cell immunotherapy in patients with EphA2-positive malignant glioma, but the trial was terminated for unknown reasons (NCT02575261). Another clinical trial was conducted, recruiting patients with recurrent EphA2-positive glioblastoma treated with EphA2-CAR-T cells (NCT03423992). Preliminary results on three patients indicated disease progression in two participants with toxicity in several organs, showing that infusions of EphA2-CAR-T cells were not well tolerated and had low clinical efficacy. However, this trial was recently concluded, and further results are needed [129]. In a recent study, Zhang et al. developed EphA2-CAR-T cells to inhibit tumor growth in prostate cancer cells, where EphA2 is highly expressed. The killing effect of EphA2-CAR-T cells was demonstrated both in vitro and in vivo using DU145 tumor xenograft mice models [130]. The same approach has been applied in breast cancer, with CAR-T cells targeting EphA2 and IL-13Rα2 effective in Her-2-enriched and triple negative breast cancer subtypes [131]. Despite emerging as a promising strategy for solid tumors, CAR-T cell therapy faces several challenges, including toxicity in normal tissues, limited efficacy, poor stability, and an immunosuppressive tumor microenvironment [131]. In a recent study, the authors used two different sets of CARs targeting different epitopes of EphA2 and tested in vitro and in vivo efficacy in glioblastoma. These CAR-T cells exhibited good antitumor activity, which was associated with upregulation and release of multiple cytokines such as interferon-γ (IFN-γ). However, the authors also observed that CAR-T cells overexpressing IFN-γ exhibited lower antitumor activity due to upregulation of PD-L1, which binds to PD-1 and suppresses T cells activity. Indeed, PD-1 blockade boosted efficacy of EphA2-CAR-T cells in xenograft mouse models [132]. Thus, the combination of EphA2-CAR-T cell therapy with ICIs may represent a valid strategy to overcome the inhibitory tumor microenvironment and enhance CAR-T cell function.

A schematic representation of the discussed approaches is shown in Figure 3.

## 5. Future Perspectives and Conclusions

Preclinical and clinical evidence support the notion that EphA2 can act as either an oncogene or oncosuppressor, depending on cellular context and availability of cognate ligands. The alterations involving EphA2 in cancer require a very careful contextual analysis that considers multiple molecular interactions and the specific tumor microenvironment. Overall, EphA2 has an impact on major cancer hallmarks such as cell migration, chemoresistance, vasculogenesis, and immune response. Compared to other RTKs, EphA2 acts through multiple cellular mechanisms, including ligand-dependent and -independent signaling, complex receptor/receptor interactions, and multiple interplays with adhesion molecules, UPS system, and soluble growth factors. Thus, understanding the complexity of EphA2 action is particularly challenging. Since post-transcriptional regulatory mechanisms play a major role, it is crucial to fully understand EphA2 modifications for the development of successful therapeutic strategies. Standard therapeutic approaches such as monoclonal antibodies and small molecules have a significant limitation in their specificity of action, as they often recognize binding sites on multiple RTKs, limiting their effectiveness and leading to the activation/suppression of pathways associated with drug resistance. PROTACs are among the new technologies aimed at overcoming these limitations through targeted protein degradation. PROTACs offer the possibility to act on undruggable molecules or those particularly difficult to target, such as EphA2, whose biological complexity severely limits therapeutic options. Other innovative approaches that offer greater specificity include drugs/toxins-conjugated bicyclic compounds and nanoparticles, which are delivered directly to tumor cells via recognition of EphA2. This approach aims to optimize the efficiency, specificity, drug payload, stability, and biocompatibility of the targeting. A similar mechanism is used by CAR-T cell therapy, and results of clinical trials will be available soon. Future research should focus on optimizing therapeutic strategies targeting EphA2, while also considering its biological role and expression in healthy tissues. Standard treatments often lead to off-target effects, but emerging therapeutic approaches are designed to significantly reduce these adverse events, resulting in lower overall toxicity.
